# Antidiabetic and Antihyperlipidemic Activities of the Leaf Latex Extract of* Aloe megalacantha* Baker (Aloaceae) in Streptozotocin-Induced Diabetic Model

**DOI:** 10.1155/2019/8263786

**Published:** 2019-04-23

**Authors:** Workineh Woldeselassie Hammeso, Yohannes Kelifa Emiru, Kefyalew Ayalew Getahun, Wubayehu Kahaliw

**Affiliations:** ^1^School of Pharmacy, College of Health Sciences, Mizan-Tepi University, Mizan Teferi, Ethiopia; ^2^School of Pharmacy, College of Medicine and Health Science, University of Gondar, Gondar, Ethiopia

## Abstract

**Background:**

Diabetes mellitus has become a major public health and economic problem across the globe. The inadequacies, as well as serious adverse effects associated with conventional medicines, led to a determined search for alternative natural therapeutic agents. The leaf latex extract of* Aloe megalacantha *has been used for the management of diabetes mellitus in Ethiopian folk medicine. This study aimed to evaluate the antidiabetic and antihyperlipidemic effects of the leaf latex extract of* A. megalacantha *in streptozotocin- (STZ-) induced diabetic model.

**Methods:**

The experimental diabetes was induced in Swiss albino mice by the administration of a single dose of STZ (150 mg/kg), intraperitoneally. The leaf latex extract of* A. megalacantha *at three different doses (100, 200, and 400 mg/kg) was administered for a period of 14 days. Fasting blood glucose levels (BGLs) were measured by glucose-oxidase and peroxidase reactive strips. After fourteen days, mice from all groups fasted and the blood was collected through puncturing the retroorbit of the eyes under mild anesthetic condition. The collected blood sample was used to determine serum biochemical parameters such as total cholesterol (TC), triglycerides (TG), low-density lipoprotein (LDL), very low-density lipoprotein (VLDL), and high-density lipoprotein (HDL) cholesterol. The statistical analysis of results was carried out using one-way analysis (ANOVA) followed by post hoc multiple comparison tests.

**Results:**

Oral administration of* A. megalacantha *leaf latex extract at doses of 100, 200, and 400 mg/kg daily for 14 days results in a significant (*p < 0.05*) decrease in fasting BGL as compared to negative control STZ-induced diabetic mice. The leaf latex has significantly reduced the level of TC, TG, and LDL, VLDL cholesterol while a significant (*p < 0.05*) HDL cholesterol increment was observed.

**Conclusions:**

The findings of the present investigation indicated that the leaf latex of* A. megalacantha *possessed significant antihyperglycemic and antihyperlipidemic potential which may prove the claimed use of the plant in amelioration of diabetes and associated complications in Ethiopian folk medicine.

## 1. Background 

Diabetes mellitus (DM) is a chronic metabolic disorder characterized by high level of glucose in the blood resulting from a relative or absolute deficiency of insulin action [1]. DM affects a population of approximately 424.9 million adults (aged 20-79) worldwide in 2017 [2]. This disease is associated with micro- and macrovascular complications which lead to the development of disability and life-threatening medical conditions [3]. Hyperglycemia, hyperlipidemia, and oxidative stress are the main important characters of DM and represent a major risk factor for the development of complications of diabetes [4, 5]. To date, the available therapy for diabetes includes insulin and various oral antidiabetic agents such as sulfonylureas, thiazolidinediones, and *α*-glucosidase inhibitors. These drugs are used as monotherapy or in combination to achieve better glycemic control [5, 6].

Good glycemic control delays the development or progression of diabetic complications but does not completely restore diabetic complications. Together with the aforementioned limitation, each of the above antidiabetic drugs is associated with a number of serious adverse effects. Therapeutic alternatives comprised of antihyperglycemic, antihyperlipidemic, and antioxidant activities with proven long-term safety should be targeted in a clinical setting for patients with coexisting diabetes and metabolic disorders [7, 8]. Hence, antidiabetic discovery has shifted its focus to medicinal plants to offer new promising efficient drugs with minimal adverse effects and lower costs [9]. Natural products play a major role in the discovery of new therapeutic agents and have received much attention as sources of bioactive substances including antioxidants, hypoglycemic, and hypolipidemic agents [5]. Of the several medicinal plants used in the local treatment of DM in Ethiopia,* Aloe megalacantha *is one of those plants used to manage diabetes.

*A. megalacantha* belongs to family Aloaceae. The plant grows on rocky hillsides and sandy alluvial plains at an altitude ranging from 1100 to 1850 meters and is found in Bale and Harerge floristic regions of Ethiopia [10]. The plant has been commonly used for the treatment of diabetes, wounds, colon cleaner, malaria, urine retention, dandruff, and impotence in Ethiopian folk medicine [11, 12]. Pharmacologically, this plant has verified* in vitro* cytotoxic effect [13]. According to the recent findings [14, 15], substances with cytotoxic activities are therapeutically recommended for antidiabetic activity. To the best of the authors' knowledge, there are no previous scientific reports on the antidiabetic activity of the leaf latex of* A. megalacantha. *Therefore, this study was undertaken to investigate the antidiabetic and antihyperlipidemic activities of the leaf latex of* A. megalacantha* in Streptozotocin- (STZ-) induced diabetic model.

## 2. Methods

### 2.1. Chemicals

STZ and glibenclamide (Alfa Aesar, Great Britain), 2,2-Diphenyl-1-picrylhydrazyl (DPPH) (Sigma-Aldrich, Germany), ascorbic acid (S.D. Fine Chemical Limited, India), glucose standard strip/kits, and glucometer (*i*-QARE DS-W Alliance International CO., Ltd Taiwan). All other chemicals and reagents used are of analytical grade.

### 2.2. Plant Material

The leaf latex of* A. megalacantha* was collected from Seharti Samre Woreda located about 57 km South West of Mekelle, the capital city of Tigray Region, and 820 km North of Addis Ababa, Ethiopia, in December 2017. The botanical identification and authentication of the plant material were performed by Mr. Abiyu Enyew (botanist) and voucher specimens (WWH-001) were deposited in Herbarium of Biology Department, Faculty of Natural and Computational Science, University of Gondar.

### 2.3. Experimental Animals

Healthy male Swiss albino mice (weighing 20-30 g and age of 8-12 weeks) were purchased from Ethiopian Health and Nutrition Research Institute (EHNRI), Addis Ababa, and kept at the animal breeding house of Department of Pharmacology, University of Gondar. The animals were kept in polypropylene cage (6-10 animals per cage), under standard laboratory conditions (at room temperature, and with a 12 h light-dark cycle), and allowed free access to the standard pelleted diet and water* ad libitum. *Before the initiation of the experiment, the animals were acclimatized to the laboratory conditions for seven days. All experiments were performed according to animal care and welfare guidelines [16]. The experiment protocol was approved by the ethical review committee of the School of Pharmacy, University of Gondar. Finally, at the end of the experiment period animals were sacrificed by cervical dislocation.

### 2.4. Extraction

The extraction and preparation process of the plant material was done as per the procedure described by Desta* et al.* [17] Briefly, matured, healthy, and fresh leaves of* A. megalacantha *were selected and washed in running tap water to remove all dirty matter and then the latex was collected by cutting the leaves transversally near the base and the latex from the leaves was eluted by gravity in sterile equipment by keeping the leaves at 45 to 90 degrees. The elution process was closely monitored to avoid mixing of the latex with gel from the cut leaves. It was then left in open air for one week to allow evaporation of water. Finally, a yellow-brown color dried leaf latex extract powder was obtained with a percent yield of 33.33% (W/V) and kept in a refrigerator until it is used for further study.

### 2.5. Acute Oral Toxicity Test

Acute oral toxicity test was performed as per limit test recommendations of the Organization for Economic Cooperation and Development (OECD) No 425 Guideline [18]. Nonpregnant and healthy young adult female Swiss albino mice (age of 8-12 weeks, weighing 20-30 g) were employed for this test. On day one, a mouse fasting for 3-4 h was given 2000 mg/kg of the extract dissolved in distilled water (DW) orally by using oral gavage. The mouse was observed for physical or behavioral changes at least once during the first 30 min, periodically for 24 h, with special attention during the first 4 h. Following the results from the first mouse, the other four mice were recruited and fasted for 3 to 4 h and were administered a single dose of 2000 mg/kg and were observed in the same manner. These observations continued for further 14 days for any signs of overt toxicity.

### 2.6. Grouping and Dosing of Animals

Swiss albino male mice were used in this study based on previously published reports which revealed that female mice were less sensitive to STZ than males and were also associated with diminished survival rate due to severe induction of diabetes by STZ [19]. Animals were divided randomly into control and treatment groups which comprise six mice in each group. The negative control group received vehicle only, the positive control group received a standard drug glibenclamide 5 mg/kg, and the remaining treatment groups were treated with three different doses (100, 200, and 400 mg/kg) of leaf latex extract of* A. megalacantha*. The doses of the extract were determined based on acute oral toxicity study. The middle dose 200 mg/kg is one-tenth of the limit dose (2000 mg/kg), the higher dose 400 mg/kg is twice the middle dose, and the lower dose 100 mg/kg was calculated as half of the middle dose. Glibenclamide 5 mg/kg was selected based on earlier studies [20, 21]. The blood sample was collected from the tail vein of mice. Fasting blood glucose level (BGL) was determined using a glucometer and each sample was measured in triplicate and then averaged.

### 2.7. Induction of Experimental Diabetes

Male Swiss albino mice fasted for 12-14 h, and weights were recorded prior to the induction of diabetes. Experimental diabetes was induced by a single intraperitoneal injection of 150 mg/ kg of STZ, freshly dissolved in 0.1 M citrate buffer (pH = 4.5) [22, 23]. Then the solution was immediately administered intraperitoneally to each mouse. Thirty minutes after the injection, the mice were allowed free access to food and water. After 6 h STZ injection, mice were given a 5% dextrose solution for the next 24 h [24, 25]. The development of diabetes was confirmed after 3 days of the STZ injection and mice with fasting BGL greater than 200 mg/dl were considered as diabetic and were selected for the experiments [26].

### 2.8. Hypoglycemic Test in Normoglycemic Mice

Mice fasted for 4–6 h and were then randomly divided into five different groups (n = 6 animals per group). The mice were treated according to their respective grouping. The blood sample was then collected from tail veins of each mouse to determine BGL at 0, 1, 2, 3, and 4 h after treatment [21].

### 2.9. Effect of* A. megalacantha *Extract on Postprandial Glycemia in Nondiabetic Mice

Male mice fasted for 4-6 h and were divided randomly into five groups which comprise six animals each group and baseline BGL was recorded. Thirty minutes after treatment, all mice were loaded with 2 g/kg glucose solution [27]. A blood sample was collected from the tail tips of the animals to determine BGL immediately after treatment at 30, 60, 120, and 180 mins [28] following glucose administration.

### 2.10. Antihyperglycemic Activity of* A. megalacantha *Leaf Latex Extracts in Diabetic Mice

After successfully developing the diabetes animals were divided into six groups and each group contains six mice. Group I: normal control mice administered vehicle only; Group III: diabetic control mice administered vehicle only; Group III: tested mice administered glibenclamide 5 mg/kg; Group IV-VI: tested mice administered* A. megalacantha* at doses of 100, 200, and 400 mg/kg, respectively. All groups received treatments once daily for 14 days. The fasting BGL and body weight were determined at 0, 7th, and 14th days [28].

### 2.11. Measurement of Serum Lipid Profiles

At the termination of treatment, i.e., 14 days, animals were deprived of food overnight. The lipid parameters such as total cholesterol (TC), triglycerides (TG), low-density lipoprotein (LDL), very low-density lipoprotein (VLDL), and high-density lipoprotein (HDL) cholesterol were evaluated using automated chemistry analyzer [28].

### 2.12. *In Vitro* Antioxidant Activity

The free radical scavenging activity of the plant extract was determined* in vitro* by DPPH assays according to the method described earlier [29]. Aliquots of 30 *μ*L of a methanolic solution containing various concentrations (1000, 500, 250, 125, and 62.5 *μ*g/ ml) of the crude extract or ascorbic acid (a reference compound) were added to 3 ml of a 0.004% MeOH solution of DPPH. The reaction mixture was shaken well and incubated in the dark for 30 min at room temperature. Then the absorbance was measured by using a spectrophotometer at 517 nm and percent inhibition was calculated. Each sample was measured in triplicate and averaged. The IC_50_ value denotes the concentration of the sample required to scavenge 50% DPPH radicals. The scavenging activity was estimated based on the percentage of DPPH radical scavenged as in the following equation:

(1) Scavenging activity %=A  cont.  –  A  testA  cont.x  100

where A cont. is the absorbance of control reaction and A test is the absorbance in the presence of extract.

### 2.13. Preliminary Phytochemical Screening

Standard phytochemical screening tests of the extract were carried out for the presence or absence of secondary metabolites such as alkaloids, steroids, terpenoids, phenols, flavonoids, tannins, anthraquinones, and saponins by using the methods as described [30, 31].

### 2.14. Statistical Analysis

All the results were expressed as mean ± standard error of means (SEM) for six mice in each group. Statistical analysis was performed by using statistical package for social sciences (SPSS) version 21.0 software. The differences between treated and untreated groups were assessed by one-way analysis of variance (ANOVA), followed by Tukey's multiple comparison tests. The result was considered significant when* p *< 0.05.

## 3. Results 

### 3.1. Acute Oral Toxicity Test

In acute oral toxicity study,* A. megalacantha *treated animals did not show any changes in their behavioral patterns and all mice survived during the whole experimental period. Hence, the median lethal dose (LD_50_) of the extract is said to be greater than 2000 mg/kg, suggesting a good safety margin.

### 3.2. Hypoglycemic Test in Normoglycemic Mice

The effect of* A. megalacantha *on BGL in the normal healthy mice is summarized in Table 1. Pretreatment with GL5 revealed a significant (*p* < 0.05 &* p* < 0.001) reduction in BGL starting 1 h when compared to negative control. Likewise, the extract also significantly (*p *< 0.05, p < 0.01 &* p* < 0.001) reduced the BGL starting 2 h onwards when compared to negative control group. Intragroup analysis, on the other hand, revealed that the leaf latex extract at a dose of 100 mg/kg produced significant (*p *< 0.001) reduction at 2nd (35.51%), 3rd (39.66%), and 4th (42.32%) in BGL compared to the baseline (t = 0), whereas glibenclamide (5 mg/kg) showed significant (*p* < 0.001) reduction in BGL at 1st (38.42%), 2nd (48.85%), 3rd (52.25%), and 4th (57.49%) compared to the baseline (t = 0). By contrast, DW10 does not produce any significant reduction in BGL across all time points compared to the baseline (t=0).

### 3.3. The effect of* A. megalacantha* Extract on Postprandial Glycemia in Nondiabetic Mice

Administration of glucose (2 g/kg) to the mice produced significant (*p* < 0.001) increase in BGL 30 min following one hour after glucose loading, confirming the induction of hyperglycemia. The extract with three doses (100, 200, and 400 mg/kg) showed a significant reduction in BGL from 60 min onwards when compared to the control group. By contrast, mice treated with GL5 showed a significant reduction in BGL starting from 30 minutes onwards compared to the negative control group. Furthermore, no apparent difference was noted when the different doses of the extract were compared with each other as well as with the positive control at all time points (Table 2).

### 3.4. Antihyperglycemic Activity of* A. megalacantha *in STZ-Induced Diabetic Mice

STZ-induced diabetic mice showed significant (*p* < 0.001) differences in BGL compared to normal control. Treatment with all doses of the leaf latex extract showed significant (*p *< 0.05 &* p *< 0.001) reduction in the BGL at the 7th and 14th days, respectively, compared to diabetic control. Similarly, GL5 treated group revealed significant (*p* < 0.001) reduction in BGL at the 7th and 14th days. Within group analysis indicated that treatment with AM100, AM200, and GL5 resulted in significant reduction in BGL at the 7th day. By contrast, AM400 failed to show reduction of BGL at the 7th day. The maximum reduction in fasting BGL was attained at the 14th day, 23.18%, 22.2%, 23.91%, and 25.22%, respectively, for AM100, AM200, AM400, and GL5 (Table 3).

### 3.5. Effect of the Leaf Latex Extract of* A. megalacantha *on Body Weight

The effects of the leaf latex extract on body weight in diabetic mice are shown in Table 4. All groups prior to extract administration (0 day) showed no apparent difference in body weight compared to normal control group. Significant body weight gain was recorded for AM200 (*p* < 0.05), AM400 (*p* < 0.01), and GL5 (*p* < 0.05) at the 7th day of treatment compared to diabetic control group. All doses of the extract and GL5 showed a significant (*p* < 0.001) improvement in body weight at the 14th day when compared to diabetic control. By contrast, the body weight of the diabetic control group was significantly decreased (*p *< 0.01) at the 14th day compared to a normal control group.

### 3.6. Effects of* A. megalacantha on* Serum Lipid Profile Levels

After the induction of diabetes and subsequent treatment with either leaf latex extract or glibenclamide, there was a significant increase of serum total cholesterol and triglycerides and a significant decrease in HDL cholesterol in diabetic mice when compared to normal mice. The results showed that administration of the latex significantly decreased (*p* < 0.05) the levels of cholesterol and triglycerides (Table 5). HDL cholesterol level was also improved in diabetic mice after 14 days of treatment. In addition, glibenclamide also improved the lipid profiles in diabetic mice.

### 3.7. *In Vitro* Antioxidant Activity

**Figure 1:**
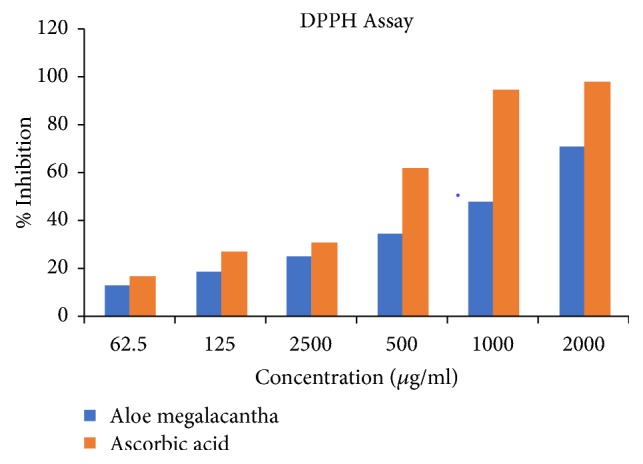
Free radical scavenging activity of the leaf latex extract of* Aloe megalacantha.*

Figure 1 illustrates the radical scavenging effect of the leaf latex extract of* A. megalacantha*. The IC_50_ of the latex was found to be 11.51 *μ*g/ml while that of ascorbic acid was 5.62 *μ*g/ml. The percent radical scavenging rate of the extract was increased in a concentration-dependent manner.

### 3.8. Phytochemical Screening

Preliminary phytochemical screening of the leaf latex extract of* A. megalacantha *showed the presence of alkaloids, flavonoids, terpenoids, tannins, phenolic compounds, saponins, and anthraquinones (Table 6).

## 4. Discussion 

The present investigation discusses the antidiabetic and antihyperlipidemic potential of the leaf latex of* A. megalacantha* in STZ-induced diabetic mice. The use of STZ to induce DM in rodent models is widely accepted and STZ-induced diabetes is reported to resemble human DM [32] which is characterized by glycosuria, hyperglycemia, polyphagia, polydipsia, and body weight loss when compared with normal rodents [33]. Glibenclamide is often used as a standard antidiabetic drug in STZ-induced moderate diabetes to compare the antidiabetic effects of a variety of bioactive compounds [34].

In present work, administration of the leaf latex of* A. megalacantha* at all tested doses (100, 200, and 400 mg/kg) resulted in a significant hypoglycemic effect in normoglycemic mice. This finding in line with Moniruzzaman et al. [35] reported the hypoglycemic effect of the related species* Aloe vera *through its insulin secretagogues potential. The hypoglycemic effect of glibenclamide was evident due to the stimulation of insulin release from pancreatic *β*-cells and inhibition of glucagon secretion [34]. The leaf latex might possess an insulin-like effect or stimulate insulin secretion from *β*-cells. Compounds of a natural product such as flavonoids and tannins isolated from medicinal plants are reported to stimulate insulin secretion from pancreatic *β*-cells [36]. Since these active constituents exist in* A. megalacantha*, the probable mechanism of action of plant latex is similar to glibenclamide.

The oral glucose tolerance test (OGTT) was performed to determine the disturbance of glucose metabolism [37]. In this study,* A. megalacantha* treated mice show a reduction in BGL within 60 min of glucose loading. This indicates that mice treated with latex have better glucose utilization capacity. Postprandial glucose lowering ability of the latex may be attributed to inhibition of glucose absorption, stimulation of peripheral glucose utilization, decrease in glycogenolysis, and gluconeogenesis [38]. Thus, oral glucose tolerance potential of the plant implies the benefit of the latex in the prevention of hyperglycemia-related complications of diabetes [28, 39].

In the present work, oral administration of* A. megalacantha* leaf latex at doses of 100, 200, and 400 mg/kg for 14 days in STZ-induced diabetic mice significantly reduced fasting BGL. Similarly, the study done by Demoz et al. [39] has reported BGL activity of the leaf latex of* Aloe camperi*. Moreover, Gundidza et al. [40] reported the antidiabetic effect of* Aloe excelsa* on male albino rats. Aloe species have been exploited for various medicinal values because of their ability to express various phytochemical constituents such as flavonoids, tannins, and terpenoids [17]. Tanaka et al. [41] have reported that* Aloe vera* gel and its derived phytosterols have a long-term BGL control effect and would be useful for the treatment of type 2 DM. Isolated phytosterols of this plant, for instance, lophenol, 24-methyl-lophenol, 24-ethyl-lophenol, cycloartanol, and 24-methylene-cycloartanol, have shown statistically significant decreases of 15 to 18% in HbA1c levels in diabetic mice. In addition, isolated antidiabetic polysaccharides, arboran A and arboran B, are reported from* Aloe arborescens* [42].

Many scholars have explained the antidiabetic mechanisms of Aloe. The first explanation is the potent antioxidant effect of aloe extract [43–45]. The present findings strongly supported the antioxidant potential of aloe, where it was found to scavenge free radicals. Since oxidative stress is involved as a causative factor in the pathogenesis of diabetes, recent approaches focus on the role of oxidative stress in pancreatic *β*-cells damage [46, 47] and hence antioxidants like aloe may have a true antidiabetic effect via antioxidant potential. Another previously suggested antidiabetic action of Aloe is through inhibition of carbohydrate metabolizing enzymes like pancreatic *α*-amylase [48]. This action decreases starch breakdown and offers good postprandial glycemic control. Furthermore, isolated sterols of Aloe, *β*-sitosterol, campesterol, and stigmasterols have been found to reduce the absorptions of cholesterol from the gut by competing for the limited space for cholesterol in mixed micelles [49, 50]. Because the test plant belongs to the same genus, the presence of such active constituents in* A. megalacantha* also may be envisaged. Hence, the antidiabetic action of the plant latex may be due to prevention of oxidative stress, inhibition of carbohydrate metabolizing enzymes, and reduction of absorptions of cholesterol from the gut and also could be due to the stimulation insulin release from residual *β*-cells or protection of functional *β*-cells from further atrophy [51, 52] by antidiabetic active constituents that may act individually or synergistically [53].

STZ-induced diabetes is associated with weight loss in mice due to protein wasting in a situation of unavailability of carbohydrate for utilization as an energy source [54]. Treatment of* A. megalacantha *for 14 days significantly improved glycemic control which prevents the loss of body weight. The increase in body weight might be attributed to structural protein synthesis or improvement of glycemic control [55].

In diabetes, the occurrence of marked hyperlipidemia may be a consequence of the uninhibited actions of lipolytic hormones on the fat depots and increase in mobilization of fatty acids from fat tissue [56]. Diabetic hyperlipidemia is featured with enhanced TG, TC, LDL, and VLDL and decreased HDL cholesterol level. These changes impose an increased risk for coronary heart disease in patients with DM. LDL-C positively and HDL-C negatively correlate with the risk of cardiovascular disease [56, 57]. The present study indicated the lowering of lipid parameters such as TG, TC, LDL, and VLDL and elevation of HDL cholesterol level in STZ-induced diabetes by the administration of* A. megalacantha. *HDL cholesterol plays a crucial role in protecting against cardiovascular disease because of its role in the transportation of excess cholesterol out of the body.

The results of* in vitro* DPPH scavenging assay suggest the antioxidant capacity of* A. megalacantha. *This result agrees with Asamenew et al. [58] findings from* A. Harlan. *Antioxidant activity is correlated with the phenolic and flavonoid compounds [59]. This effect is thought to be due to the strong ability of the latex to act as a donor of hydrogen atoms or electron.

Preliminary phytochemical screening of the leaf latex extract of* A. megalacantha *showed the presence of alkaloids, flavonoids, terpenoids, tannins, phenolic compounds, saponins, and anthraquinones. The significant antidiabetic, antihyperlipidemic, and antioxidant activity of the leaf latex of* A. megalacantha *in this study may be attributed to the presence of these principal constituents.

## 5. Conclusions 

In conclusion, the present findings demonstrated that the leaf latex extract of* A. megalacantha *Baker is capable of exhibiting significant antihyperglycemic activities in STZ-induced diabetic mice. The plant also showed improvement in parameters such as oral glucose tolerance, body weight, lipid profile, and hypoglycemic and antioxidant activity. The results give scientific support for the use of the plant in folk medicine for the management of diabetes and its associated complications.* A. megalacantha* would be promising for further clinical studies on* Aloe* extract or extract components in the management of DM. Further studies to find out the mechanism of this plant for its antidiabetogenic effect and to identify the bioactive compounds responsible for this effect are necessary.

## Figures and Tables

**Table 1:** Effect of leaf latex extract of* Aloe megalacantha* on BGL of normoglycemic mice.

Group	Blood glucose (mg/dl) level in different time intervals (hours)				
	0 h	1 h	2 h	3 h	4 h
DW 10	121.00 ± 1.93	120.33 ± 4.42	118.83 ± 6.50	123.50 ± 8.84	124.33 ± 10.05
GL5	114.50 ± 3.84	70.50 ± 3.41^1a, 3b^	62.00 ± 3.11^3a, 3b^	54.67 ± 2.74^3a, 3b^	48.67 ± 2.82^3a, 3b^
AM100	119.33 ± 2.11	103.17 ± 8.06	76.95 ± 5.18^1a, 3b^	72.00 ± 5.16^2a, 3b^	68.83 ± 7.68^2a, 3b^
AM200	110.67 ± 3.33	90.00 ± 10.48	84.83 ± 9.02^1a^	77.33 ± 10.93^1a^	68.67 ± 9.73^2a1b^
AM400	114.83 ± 3.60	85.28 ± 12.72	71.95 ± 9.71^2a, 1b^	66.67 ± 7.50^3a, 1b^	66.72 ± 8.75^3a, 1b^

Results are expressed in mean ± S.E.M, n = 6, DW10 = distilled water 10 ml/kg; GL5 = glibenclamide 5mg/kg; AM100 = *A. megalacantha* extract 100 mg/kg; AM200 = *A. megalacantha *extract 200mg/kg; AM400 = *A. megalacantha *extract 400mg/kg;  ^a^compared with negative control group;  ^b^compared with fasting blood glucose level; c compared with glibenclamide 5mg/kg (t = 0 h); ^1^p < 0.05, ^2^p < 0.01, ^3^p < 0.001.

**Table 2:** The effects of the leaf latex of *Aloe megalacantha* on postprandial glycemia in nondiabetic mice.

Group	Blood glucose level(mg/dl)				
	0 min	30 min	60 min	120 min	180 min
DW10	103.67 ± 3.49	183.33 ± 9.16	155.17 ± 6.91^1c^	109.00 ± 3.76^3c^	101.83 ± 3.60^3c^
GL5	96.67 ± 1.89	123.56 ± 10.8^1a^	77.50 ± 1.68^3a, 3c^	67.67 ± 2.43^2a2b3c^	61.67 ± 2.44^3a, 2b, 3c^
AM100	99.33 ± 3.79	135.67 ± 15.98	74.17 ± 7.55^3a, 2c^	61.76 ± 8.45^2a, 3c^	69.78 ± 7.69^2a, 3c^
AM200	101.17 ± 2.99	161.83 ± 13.93	62.17 ± 9.29^3a, 1b, 3c^	55.95 ± 7.35^3a2b3c^	62.00 ± 4.29^3a, 1b, 3c^
AM400	100.00 ± 2.74	171.17 ± 11.08	83.83 ± 6.99^3a, 3c^	75.61 ± 10.40^1a, 3c^	71.00 ± 4.21^3a, 3c^

Results are expressed in mean ± S.E.M, n = 6, DW10 = distilled water 10 ml/kg, GL5 = glibenclamide 5mg/kg, AM100 = *A. megalacantha *extract 100 mg/kg, AM200 =* A. megalacantha* extract 200mg/kg, AM400 = *A. megalacantha* extract 400mg/kg,  ^a^compared with negative control group, b compared with BGL at 0 min, c compared with BGL after 30 min,  ^d^compared with glibenclamide 5mg/kg 1p < 0.05, 2p < 0.01,3p < 0.001

**Table 3:** The effects of the leaf latex extract of *Aloe megalacantha* in STZ-induced diabetic mice.

Groups	Blood glucose level in mg/dL		
	Day 0	Day 7	Day 14
NC	101.33 ± 4.07^3a^	99.83 ± 3.20^3a^	102.33 ± 3.88^3a^
DC	264.33 ± 5.49	266.83 ± 1.13	268.83 ± 5.05
GL5	256.33 ± 4.63	219.50 ± 4.28^3a3b^	191.67 ± 1.58^3a3b3c^
AM100	265.33 ± 7.67	241.50 ± 6.55^1a1b^	203.83 ± 2.35^3a2b2c^
AM200	262.17 ± 5.29	239.50 ± 7.10^1a1b^	204.00 ± 1.96^3a3b3c^
AM400	265.50 ± 9.86	241.17 ± 6.94^1a^	202.00 ± 1.88^3a3b2c^

Results are expressed in mean ± S.E.M, n = 6; DW10 = distilled water 10 ml/kg; DC = diabetic control; GL5 = glibenclamide 5 mg/kg; AM100 = *A. megalacantha* extract 100 mg/kg; AM200 =* A. megalacantha* extract 200 mg/kg; AM400 = *A. megalacantha* extract 400 mg/kg; ^a^compared with diabetic control group, ^b^compared to 0 days, ^c^compared to 7 days, ^1^*p* < 0.05, ^2^*p* < 0.01, ^3^*p* < 0.001.

**Table 4:** Effects of the leaf latex extract of *Aloe megalacantha *on body weight in diabetic mice.

Groups	Body weight in gram		
	Day 0	Day 7	Day 14
NC	27.02 ± 0.87	27.83 ± 0.69	27.25 ± 0.91
DC	25.65 ± 0.84	24.83 ± 1.4	23.83 ± 0.95^2b^
GL5	24.97 ± 0.71	28.88 ± 0.55^1a^	29.63 ± 0.50^3a^
AM100	25.50 ± 1.09	27.54 ± 0.66	29.03 ± 0.90^3a^
AM200	25.33 ± 0.67	29.04 ± 0.70^1a^	29.8 9± 0.77^3a^
AM400	24.17 ± 0.79	29.72 ± 0.22^2a^	30.21 ± 0.43^3a^

All data were expressed as in mean ± S.E.M, n = 6; NC = normal control; DC = diabetic control;  ^a^compared to diabetic control,  ^b^compared to normal control, ^1^p < 0.05, ^2^p < 0.01, ^3^p < 0.001.

**Table 5:** Effect of *Aloe megalacantha *on serum lipid profiles.

Groups	STC (mg/dl)	STG (mg/dl)	HDL-c (mg/dl)	VLDL-c (mg/dl)	LDL-c (mg/dl)
NC	89.00 ± 0.966	95.83 ± 5.87	35.83 ± 1.08^3a^	19.17 ± 1.17^3a^	34.00 ± 1.67a
DC	189.33 ± 1.54^3a^	176.50 ± 2.75^3a^	24.17 ± 1.25^3a^	35.30 ± 0.55^3a^	129.86 ± 2.65^3a^
GL5	92.00 ± 2.08^3a^	99.67 ± 3.03^3a^	37.33 ± 0.49^3a^	19.93 ± 0.60^3a^	34.74 ± 2.05^3a^
AM100	171.33 ± 4.82^1a, 3b^	151.67 ± 5.32^1a, 3b^	29.83 ± 1.40^1a, 1b^	30.33 ± 1.06^1a^	111.33 ± 5.49^1a^
AM200	165.67 ± 5.24^2a, 3b^	147.67 ± 4.69^2a, 3b^	31.83 ± 1.22^3a^	29.53 ± 0.94^2a^	104.30 ± 5.08^2a^
AM400	160.83 ± 5.07^3a, 3b^	142.17 ± 6.83^3a, 3b^	34.17 ± 0.79^3a^	28.43 ± 1.37^3a^	98.17 ± 4.81^3a^

Results are expressed in mean ± S.E.M, n = 6; NC = normal control; DC = diabetic control; GL5 = glibenclamide 5mg/kg; AM100 = *A. megalacantha* extract 100 mg/kg; AM200 = *A. megalacantha *extract 200mg/kg; AM400 = *A. megalacantha *extract 400mg/kg; STC = serum total cholesterol; STG = serum triglyceride; HDL-c = high-density lipoprotein cholesterol; VLDL-c = very low-density lipoprotein cholesterol; LDL-c = low-density lipoprotein cholesterol;  ^a^compared with normal control group,  ^b^compared with diabetic control; ^1^p < 0.05, ^2^p < 0.01, ^3^p < 0.001.

**Table 6:** Phytochemical screening of leaf latex of *Aloe megalacantha*.

Phytochemicals	Tests	Results
Alkaloids	Wagner's test	+
Flavonoids	Lead acetate test	+
Phenols	Ferric chloride test	+
Tannins	Ferric chloride test	+
Steroids	Salkowski's test	-
Saponins	Foam test	+
Glycosides	Glycoside test	+
Anthraquinones	Borntrager's test	+
Terpenoids	Salkowski's test	+

(+) presence of a given secondary metabolite, (-) absence of a given secondary metabolite

## Data Availability

The datasets used and/or analyzed during the current study are available from the corresponding author upon reasonable request.

## Abbreviations

BGL: Blood glucose level
DM: Diabetes mellitus
DW: Distilled water
EPHI: Ethiopian Public Health Institute
HRERC: Health Research Ethics Review Committee
IDF: International Diabetes Federation
OECD: Organization for Economic Cooperation and Development
OGTT: Oral glucose tolerance test in mice
ROS: Reactive oxygen species
STZ: Streptozotocin
WHO: World Health Organization.
